# Electroacupuncture for Postoperative Urinary Retention: A Systematic Review and Meta-Analysis

**DOI:** 10.1155/2018/7612618

**Published:** 2018-07-26

**Authors:** Yajing Zhong, Fanzhu Zeng, Jiaying Li, Yunhua Yang, Shuxian Zhong, Yang Song

**Affiliations:** ^1^School of Nursing, Guangzhou University of Chinese Medicine, Guangzhou, Guangdong, China; ^2^Zhongshan School of Medicine, Sun Yat-sen University, Guangzhou, Guangdong, China

## Abstract

**Background:**

This systematic review aimed at summarizing and evaluating the evidence from randomized controlled trials (RCTs) which used electroacupuncture (EA) to treat postoperative urinary retention (PUR).

**Methods:**

We searched thirteen databases electronically through April 2018 without language restrictions. We included RCTs of women with PUR; other types of urinary retention or not-RCTs were excluded. Two independent reviewers extracted studies' characteristics, and disagreements were resolved by consensus. Data were pooled and expressed as standard mean difference (SMD) for continuous outcomes and odds ratio (OR) for dichotomous outcomes, with 95% confidence interval (CI).

**Results:**

We found very low to moderate level of evidence that effects of less than or equal to a week were statistically significant: therapeutic effect improved (OR=4.21; 95%CI [3.04, 5.83]; P<0.00001), residual urine volume decreased (SMD=-13.24; 95%CI [-15.70, -10.78]; P<0.00001), bladder capacity increased (SMD=0.56; 95%CI [0.30, 0.83]; P<0.0001), and urinary flow rate improved (SMD=0.91; 95%CI [0.64, 1.18]; P<0.00001). Effect over a week was statistically significant as well. Therapeutic effect improved (OR=8.29; 95%CI [2.91, 24.25]; P<0.0001), residual urine volume decreased (SMD=-1.78; 95%CI [-2.66, -0.89]; P<0.0001), bladder capacity (SMD=0.92; 95%CI [0.61, 1.23]; P<0.00001) and urinary flow rate (SMD=1.69; 95%CI [0.59, 2.79]; P=0.003) increased, and first urination after surgery was earlier (SMD=-0.92; 95%CI [-1.37, -0.46]; P<0.0001), compared with physical exercise, medication, or no treatment.

**Conclusion:**

The efficacy and safety of EA on key outcomes in women with PUR are statistically significant, but the level of most evidence was very low or low. More large-scale, long-term RCTs with rigorous methodological quality are needed.

## 1. Introduction

Postoperative urinary retention (PUR) is defined as the inability to void with a full bladder and is a well-recognized and common complication of surgery and anaesthesia [1]. The occurrence of urinary retention after anaesthesia and surgery ranges between five to seventy percent, depending on the types of surgeries and the criteria used to define urinary retention [1]. Although the true incidence is unknown and diagnosis is often arbitrary, it is associated with significant patient morbidity, delayed hospital discharge, and need for additional follow-up [2]. The risk of urinary retention increases with advancing age and is dependent on other factors such as the types of surgeries and anaesthesia and duration of time under anaesthesia [1, 3–7]. PUR can complicate any surgical procedure and is not limited to patients with preexisting urinary symptoms [8]. Although often regarded by clinicians as a trivial or minor complication, urinary retention can be a significant source of anxiety and discomfort [9]. Retention prolongs hospital stay increases costs and may result in significant morbidity [10, 11]. In senior patients, urinary retention can be associated with restlessness, confusion, and potential development of delirium [12, 13].

Preoperative screening and assessment to identify patients at risk of this complication are crucial [14]. Given the adverse results associated with urinary retention, patients at increased risk of PUR should be identified before surgery and the condition should be treated in a timely manner following surgery [2, 15, 16]. Recognition of the risk factors that contribute to its development in the surgical patients is vital to ensure prompt, appropriate care, and treatment interventions [2].

To resolve postoperative urinary retention, doctors often use catheterization to relieve pain for patients. Urinary catheterization is an invasive procedure with potential morbidity and mortality [8]. Urinary tract infections account for at least thirty-five percent of all hospital-acquired infections, and eighty percent of these infections are caused by insertion of a urethral catheter [17–19]. Other potential complications include tissue trauma, blockage, and encrustation [20]. Catheterization can result in urethral strictures which demand additional surgery and may be associated with a higher hospital mortality rate in vulnerable patient populations [21, 22]. Despite best nursing care, for each day an indwelling urethral catheter remains in place the risk of infection increases by three to ten percent [23]; the longer the urethral catheter remains in place, the risk increases cumulatively [24]. For patients who have been catheterized for more than seven to ten days, the risk of catheter-related urinary tract infections may increase by fifty percent. With these risks in mind, this invasive procedure can only be performed when it is absolutely necessary [24]. Therefore, we need to seek other safer and more effective therapies to relieve the symptoms.

Acupuncture originated in ancient China has been reported in the literature that it may be an effective treatment option [25, 26], especially the EA, and is reported and recommended as a supplementary or alternative treatment for various diseases, including PUR [27]. EA is a form of acupuncture with electrical impulses passing through the needles to stimulate acupoints [28, 29]. Although the mechanism of EA is still not clear, EA has been widely used clinically by practitioners of traditional Chinese Medicine to treat PUR in China and the efficacy is satisfactory [30]. Acupuncture has a good therapeutic effect, and EA has been found to speed up the recovery of urinary retention, which was safe and mild [27]. EA can significantly reduce residual urine volume. The therapeutic effect is better than that of medication and PFMT. The long-term curative effect needs to be further studied and evaluated [30].

There are many clinical trials on the efficacy of EA for PUR but only two related meta-analyses, which used acupuncture and moxibustion as interventions [31, 32]. No systematic reviews and meta-analyses on EA for PUR especially, hence, the effectiveness and safety of this treatment, remain unclear. We aim to assess the effectiveness and safety of EA for PUR and provide evidence for further enhancing the clinical therapeutic effect on patients with PUR. The study may answer whether EA was exactly safe and effective for patients with PUR?

## 2. Methods

This systematic review and meta-analysis is reported in accordance with the Preferred Reporting Items for Systematic Reviews and Meta-Analyses (PRISMA) Statement [33] and was registered at International Prospective Register of Systematic Reviews (number CRD42018092574).

### 2.1. Literature Search Strategy

We systematically searched databases for relevant studies published through April 2018, comprising six international, four Chinese, one Korean, one Japanese, and one Russian databases. Including PubMed, EMBASE, the Cochrane Central Register of Controlled Trials (CENTRAL), Web of Science, Clinical Trails.gov.cn, BIOSIS Previews, China National Knowledge Infrastructure Database (CNKI), Chinese Biological Medicine Database (CBM), VIP Database, WanFang Digital Periodicals Database (WFDP), Korean Citation Index (KCI), Japan Science and Technology Information Aggregator, Electronic (J-STAGE), and Russian Science Citation Index (RSCI) for studies that assessed the safety and effect of EA on PUR. The search words were acupuncture (e.g., acupuncture, electroacupuncture, needle, and needles) and urinary retention (e.g., urinary retention, retention urinary, and postoperative urinary retention). We did not apply any date or language restrictions.

We used the following combined text and MESH terms for PubMed search: (((((＇＇Acupuncture＇＇[Mesh]) OR Pharmacopuncture[Title/Abstract]) OR ＇＇Electroacupuncture＇＇[Mesh]) OR ＇＇Needles＇＇[mesh]) OR Needle[Title/Abstract]) AND (((＇＇Urinary Retention＇＇[Mesh]) OR Retention, Urinary[Title/Abstract]) OR Postoperative urinary retention[Title/Abstract]) AND ((clinical[tiab] AND trial[tiab]) OR ＇＇clinical trials as topic＇＇[mesh] OR ＇＇clinical trial＇＇[pt] OR random*∗*[tiab] OR ＇＇random allocation＇＇[mesh] OR ＇＇therapeutic use＇＇[sh]). We searched the databases between inception and April, 2018.

### 2.2. Inclusion and Exclusion Criteria

#### 2.2.1. Types of Studies

All RCTs of EA for PUR were included. Nonrandomized trials, quasiexperimental studies, and observational studies were excluded. Animal studies, qualitative studies, letters, news articles, editorials, and commentaries were also excluded.

#### 2.2.2. Types of Participants

Clinical trials of participants diagnosed with PUR with painful, palpable, or percussible bladder which suppressed micturition reflex were included. The diagnostic criteria adopted in our review were based on the International Continence Society (ICS) without any age and race limit [34]. Studies of patients complaining of serious systemic or neurologic disease (diabetes, AIDS, epilepsy, etc.), urinary system infection, preoperative radiotherapy or chemotherapy, combination of serious risk such as cardiovascular, liver, kidney, and hematopoietic system, or refusal to accept acupuncture treatment were excluded, for they could have a unusual natural history.

#### 2.2.3. Types of Interventions

Studies of EA for PUR were included. Used EA alone as an intervention or with other treatments can be included. However, other acupuncture methods (nonelectroacupuncture) and dry needling not based on oriental medicine and meridian theory were excluded.

#### 2.2.4. Types of Control Groups

Conventional therapy generally used for PUR such as usual care, medication, physical therapy modalities done by the general physician or no treatment were included. However, herbal medicine as the control group could not be seen as a conventional therapy and thus was excluded.

#### 2.2.5. Types of Outcome Measures

In this study we analyzed therapeutic effect of EA, residual urine volume, time to first urination, bladder capacity, urinary flow rate, and urine output to evaluate the efficacy of EA.

### 2.3. Data Extraction

Two reviewers (YZ and FZ) independently extracted data, including the quality assessment from the retrieved studies. The titles and abstracts were reviewed and articles that did not fit the eligibility criteria were excluded. If the title or abstract appeared to meet the eligibility criteria, the full texts of the articles were obtained for further evaluation. Discrepancies were resolved in a consensus meeting or, if agreement could not be reached, they were resolved by referral to a third reviewer (YS). The independent reviewers extracted and tabulated data using a standardized data extraction form, with disagreements finally interpreted by the corresponding author (YS).

We extracted the following data from each selected study: the first author, published year, total number of participants, age, and country where the trial was conducted, course of disease, healing period, surgical approach, the details of intervention and control group, outcome indicators, and reported adverse events. If the data in a study were insufficient or ambiguous, one reviewer (YZ) contacted the corresponding author by e-mail to obtain further information. Two independent reviewers (YZ and FZ) assessed risk for bias according to the PRISMA recommendations [33].

### 2.4. Assessment for Risk of Bias

Two reviewers (YZ and FZ) independently evaluated the risk of bias among the final included studies using the risk of bias assessment tool by the Cochrane Collaboration [35]. The criteria consist of seven items including selection bias (random sequence generation and allocation concealment), performance bias (blinding of participants and personnel), detection bias (blinding of outcome assessment), attrition bias (incomplete outcome data), reporting bias (selective reporting), and other biases. Each study was evaluated as high, low, or unclear risk of bias for each item, and the assessment criteria were based on the Cochrane handbook [35]. Any disagreements between the two reviewers were resolved by discussion with the corresponding author (YS) until consensus was reached.

### 2.5. Statistical Analysis

Statistics analysis was done using the Review Manager program (Version 5.3 Copenhagen: The Nordic Cochrane Centre, The Cochrane Collaboration, 2014). We combined studies according to the type of intervention and assessed the therapeutic effect of EA, residual urine volume, first urination time, bladder capacity, maximum urinary flow rate, and urine output. Dichotomous data were summarized as OR and continuous data as SMD. Heterogeneity between studies was evaluated by using X2 (chi-squared) test with p-value of p<0.05 and I^2^ statistic. I^2^ was used to assess heterogeneity between studies, with *⩾*50% considered to indicate a substantial level of heterogeneity [36, 37]. A fixed-effect model was used when there was no significant heterogeneity between studies; otherwise a random-effect model was employed and subgroup analysis or sensitivity analysis was performed to explore heterogeneity [35]. 95%CI were calculated, and p<0.05 was regarded as statistical significant [36, 37]. If a substantial heterogeneity was detected, we explored sources of heterogeneity by subgroup analyses. Subgroup analyses were attempted according to the difference of interventions. If some factors could not be found, we did not conduct subgroup analyses or data syntheses but reported a narrative description of the included studies. We conducted a sensitivity analysis using the leave-one-out approach if there was high heterogeneity between studies. Publication bias was evaluated using a funnel plot analysis if a sufficient number of trials (ten trials) were found; it would not be done in cases of less than ten studies in a group.

### 2.6. Level of Evidence

Grading of Recommendations, Assessment, Development, and Evaluation (GRADE) was used to assess the level of evidence and summarize every outcome [38]. The level of evidence was classified as high, moderate, low, or very low. Evaluation of the level of evidence was done on the following domains: risk of bias, inconsistency, indirectness, imprecision, and publication bias. The GRADE prosoftware (version 3.6.1 for Windows, Grade Working group) was used.

## 3. Results

### 3.1. Search Results

**Figure 1:**
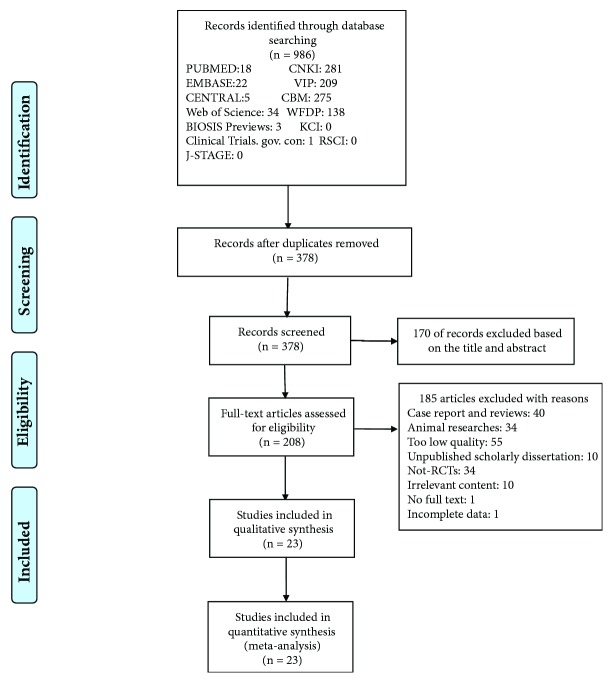
Study flow diagram.

Our database search retrieved 986 articles, 18 from PubMed, 22 from EMBASE, 5 from CENTRAL, 1 from the Clinical Trials. gov. con, 34 from Web of Science, 3 from BIOSIS Previews, 281 from CNKI, 209 from VIP Database, 275 from CBM, 138 from WFDP. We did not find related articles in KCI, J-STAGE, or RSCI. 378 records were screened after removal of duplicates. Of these, 170 were excluded after reading titles and abstracts, 40 articles were excluded because they were case reports or reviews, 34 animal experimental research were eliminated, and full texts of 134 articles were downloaded and assessed. During further evaluation, 55 were excluded for too low article quality, 10 were excluded for the reason that they were unpublished scholarly dissertations, and 34 non-RCTs were excluded as well. 5 articles were about urinary retention prevention before surgery, 1 was comparing the efficacy of EA with acupuncture, and 4 were excluded because they were published repeatedly. 1 full text could not be found and 1 article was excluded due to incomplete data. Finally, 23 RCTs were included. The flow chart of the analysis is presented in**Figure 1**.

### 3.2. Included Studies and Characteristics

The table showed the main study characteristics. 1861 patients were included in the analysis, of whom 961 (51.64%) were brought into experimental group and 900 (48.36%) were brought into control group. Although we did not set a national limit, the 23 studies were all conducted in China and published between 2004 and 2017. The age of patients among studies ranged from 19 to 75, 23 studies we included, but only 3 studies [39–41] described the indicator of body mass index (BMI). 12 studies [39, 42–52] had a course of treatment less than or equal to one week, 8 studies [40, 41, 53–58] owned a treatment course over one week, and 3 studies [59–61] did not show the treating time. 10 studies [39–41, 45, 46, 48, 49, 55–57] were performed on hysterectomy, 8 [42–44, 47, 51, 59–61] were performed on anorectal surgery, 2 [52, 58] were performed on orthopedic surgery, 1 [53] was performed on prostate surgery, 1 [50] was performed on spinal anaesthesia, and 1 study [54] did not indicate the type of surgery. All of the included studies used EA with or without other treatments as intervening measure, 8 of these studies [39, 41, 43, 45, 54, 55, 57, 58] compared EA with usual care and physiotherapy, and 10 studies [40, 42, 46–49, 51, 53, 56, 59] compared EA with medication. 5 studies [44, 50, 52, 60, 61] set EA as intervening measure while there was no intervention in the control group. Baseline characteristics among groups were reported as comparable in each study.

Studies evaluated the therapeutic effect of EA according to the reported urinary retention after treatment except for 2 [52, 58], 10 studies [39–41, 45, 51, 54–58] tested the residual urine volume, 5 studies [50–52, 54, 61] reported time to first urination, 3 studies [39, 41, 56] reported bladder capacity, 4 studies [39, 41, 56, 58] reported urinary flow rate, and 5 studies [41, 50–52, 56] reported the urine output. The number and period of treatment sessions varied in each study. A summary of the included studies in more detail is presented in**Table 1**.

### 3.3. The Assessment for Risk of Bias (Figures 2 and 3).

#### 3.3.1. Random Sequence Generation

Among the 23 studies, 6 [39–41, 50, 52, 57] used computer-programmed random sequencing, a random number table or random number generator, and were thus evaluated as low risk of bias. 12 [42–45, 47, 53, 55, 56, 58–61] did not mention the method or detail of random sequencing and were evaluated as an unclear risk of bias. Other 5 studies [46, 48, 49, 51, 54] were evaluated as high risk of bias for patients who were assigned to the experimental group or experimental group with the admission date being singular or even and the order of visit.

#### 3.3.2. Allocation Concealment

Of the 23 studies, 7 [39–41, 48, 50, 52, 57] used sealed-envelops, random list, random assignment method, or cast coins to determine the grouping of single and double numbers were given a low risk of bias based on allocation concealment, while 2 studies [49, 51] were given a high risk of bias because they randomly divided patients into two groups according to the order of visits. Others were at unclear risk of bias because they did not describe any method of allocation concealment.

#### 3.3.3. Blinding of Participants and Personnel

Due to the nature of the active control and EA, most of the studies did not perform blinding. Only in the study of Weimin Yi (2014) were the participants assessment blinded, using sham electroacupuncture as control intervention, resulting in a low risk of bias. The rest were evaluated as high risk of bias for compared EA with medication, usual nursing, physiotherapy, or no treatment.

Blinding of outcome assessment: for outcome blinding, only 1 study by Weimin Yi (2014) adopted single-blind method to evaluate the intervention measure and consider the blind effect as favorable to have a low risk of bias. We rated the other studies as having an unclear risk of bias because insufficient information was provided to determine whether investigators were blinded or not.

#### 3.3.4. Incomplete Outcome Data

3 studies [41, 50, 52] described reasons for dropouts and lost data adequately in the published reports and statistical analysis of data based on intention to treat. The rest studies did not lost numbers or data, so we considered all studies as having a low risk of bias.

#### 3.3.5. Selective Outcome Reporting

4 studies [41, 42, 57, 60] we included reported that all expected outcomes including adverse events were evaluated as low risk of bias. Study He Changhai (2010) that only listed the total results and the outcome indicators were incomplete was considered as having a high risk of bias. The rest of the studies did not register the protocol and did not mention adverse events, thus evaluated as high risk of bias as well.

#### 3.3.6. Other Sources of Bias

All studies were at low risk of bias based on lack of clear evidence to display other obvious bias.

#### 3.3.7. Analysis

Since the RCTs included in this study vary in study designs and treatment course, they need to be categorized by the types of interventions and treatment course. Studies were categorized for analysis by the types of intervention (EA with or without other treatment versus physiotherapy, medication, or no treatment) and duration of treatment.

## 4. Outcomes

### 4.1. Therapeutic Effect

**Figure 4:**
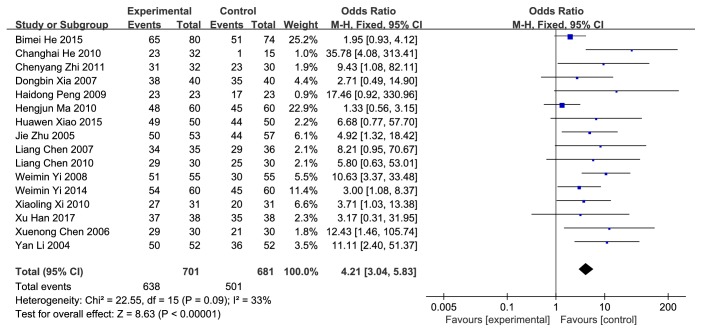
Forest plot of pooled estimation of therapeutic effect (less than or equal to a week).

**Figure 5:**
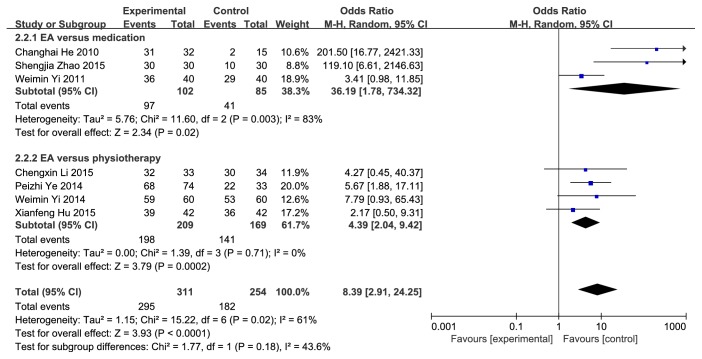
Forest plot of pooled estimation of therapeutic effect (over a week).

Primary outcome was the therapeutic effect of EA on PUR. The definition of “therapeutic effect” differed among these studies, which might have included ability to urinate after treatment, and symptoms and signs of discomfort have been improved, no need for catheterization and a residual urine volume of <100 ml. We divided the curative effect into two parts according to the treatment time less than or equal to a week and more than a week. Obtaining data from 21 studies, 16 [39, 41–51, 53, 59–61] described the efficacy which were less than or equal to a week while 7 [40, 41, 53–56] described the efficacy which were over a week. The total therapeutic effect (less than or equal to a week) had statistical significance (OR=4.21; 95%CI [3.04, 5.83]; P<0.00001; I^2^=33%), indicating obvious effect of EA with low heterogeneity (**Figure 4**). The therapeutic effect (more than a week) was statistical significant (OR=8.39; 95%CI [2.91, 24.25]; P<0.00001; I^2^=61%) with substantial heterogeneity (**Figure 5**). In order to further explore the sources of heterogeneity, we conducted a sensitivity analysis. Removing the Changhai He (2010) study, the heterogeneity reduced to 29%, and we considered that possibly the differences in surgical methods of this study caused the heterogeneity. Only this study [53] performed prostatectomy, and the rest were hysterectomy, except the Chengxin Li (2015) study did not indicate the surgical approach. The data were analyzed in subgroups according to the difference of interventions. Heterogeneity decreased when the intervention on patients was divided into 2 groups: EA versus medication and EA versus physiotherapy. 3 studies [40, 53, 56] assigned to the group EA versus medication showed a statistical significance (OR=36.19; 95%CI [1.78, 734.32]; P=0.02; I^2^=83%). Significant heterogeneity disappeared when we eliminated the Weimin Yi (2011) study, and we speculated that the reason might be that the control group in this study used acupoints injection of vitamin B12 while the other two used western medicine plus basic nursing and PFMT. 4 studies [41, 54, 55, 57] distributed to the group EA versus physiotherapy showed a statistical significance (OR=4.39; 95%CI [2.04, 9.42]; P=0.0002; I^2^=0%) without heterogeneity. The outcome of therapeutic effect was relatively robust.

### 4.2. Residual Urine Volume

**Figure 6:**
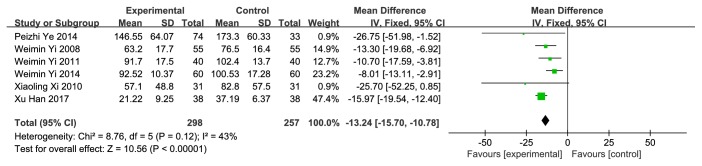
Forest plot of pooled estimation of the residual urine volume (less than or equal to a week).

**Figure 7:**
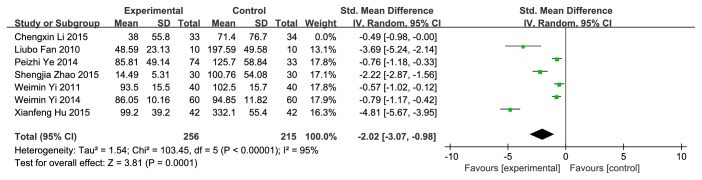
Forest plot of pooled estimation of the residual urine volume (over a week).

Data we gathered from 10 studies were divided into two parts according to the healing period. 6 studies [39–41, 45, 51, 57] were assigned to the group treating less than or equal to a week (**Figure 6**). 7 [40, 41, 54–58] were assigned to another group that treating over a week (**Figure 7**). The result (less than or equal to a week) showed statistical significance (SMD=-13.24; 95%CI [-15.70, −10.78]; P<0.00001 I^2^=43%), indicating that EA significantly reduced the residual urine volume, with the heterogeneity in a tolerable level. Another result (over a week) had statistical significance (SMD=-1.78; 95%CI [-2.66, −0.89]; P<0.0001; I^2^=95%) with considerable heterogeneity. We conducted a sensitivity analysis due to high heterogeneity; when removing the Xianfeng Hu (2015) study, the heterogeneity reduced to I^2^=85%, which might be the source of heterogeneity; though the heterogeneity was still high, the outcome was relatively robust. It was considered that the source of the heterogeneity might be that the Xianfeng Hu (2015) study used traditional Chinese medicine nursing as an adjunctive intervention with EA, including emotional care, functional exercise, and massage treatment. We tried to perform subgroup analysis to reduce heterogeneity but no obvious change of the outcome. The heterogeneity was not resolved in spite of the subgroup analysis.

### 4.3. Time to First Urination

**Figure 8:**
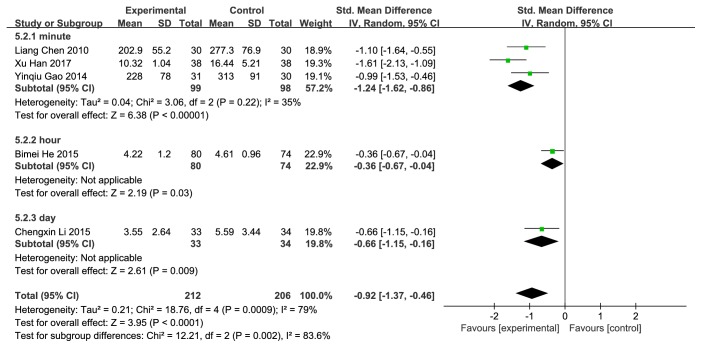
Forest plot of pooled estimation of the first urinating time.

5 studies [50–52, 54, 61] were included. The comparison of first urinating time among studies were statistically significant (SMD=-0.92; 95%CI [-1.37, −0.46]; P<0.0001; I^2^=79%); the initial urinary time of the experimental group is relatively early. We conducted a sensitivity analysis to look for instability. Removing Bimei He (2015) and Xu Han (2017) studies, the heterogeneity disappeared. However, we could not find commonalities between these two studies which were independent of other studies. 5 studies were analyzed in subgroups according to the treating time unit. 3 [51, 52, 61] were assigned to the subgroup “minute” and the heterogeneity reduced to I^2^=35%. 1 [50] in the subgroup “hour”, 1 [54] in the subgroup “day” (**Figure 8**).

### 4.4. Bladder Capacity

**Figure 9:**
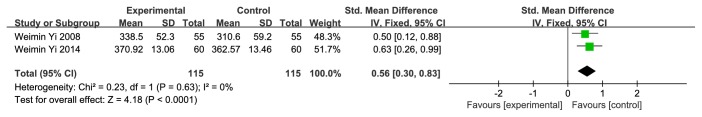
Forest plot of pooled estimation of the bladder capacity (less than or equal to a week).

**Figure 10:**
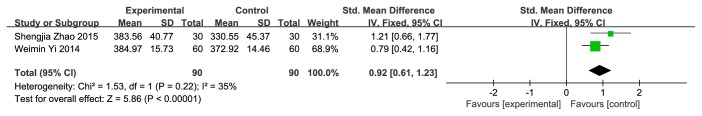
Forest plot of pooled estimation of the bladder capacity (over a week).

3 studies [39, 41, 56] which described bladder capacity were divided into two groups according to their treating time. 2 studies [39, 41] distributed to the group treating less than or equal to a week showed that the bladder capacity of the experimental group is larger than that of the control group (SMD=0.56; 95%CI [0.30, 0.83]; P<0.0001; I^2^=0%) without heterogeneity (**Figure 9**). 2 studies (Weimin Yi et al., 2014; Shengjia Zhao et al., 2015) distributed to the group treating more than a week showed statistical significance (SMD=0.92; 95%CI [0.61, 1.23]; P<0.00001; I^2^=35%) with low heterogeneity (**Figure 10**).

### 4.5. Urinary Flow Rate

**Figure 11:**
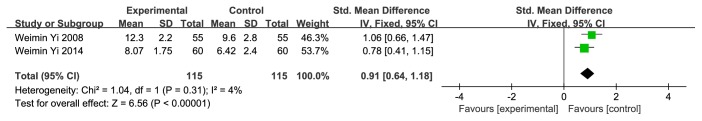
Forest plot of pooled estimation of the urinary flow rate (less than or equal to a week).

**Figure 12:**
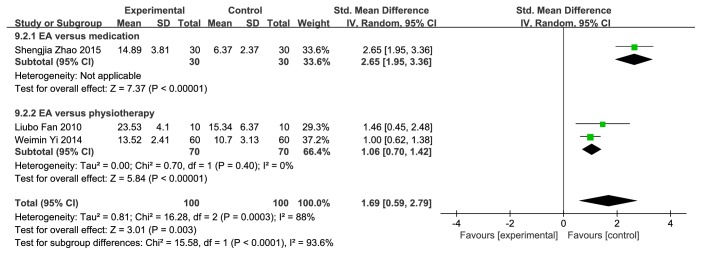
Forest plot of pooled estimation of the urinary flow rate (over a week).

4 studies [39, 41, 56, 58] which reported urinary flow rate were divided into two groups due to different treatment duration. 2 studies [39, 41] were treated less than or equal to a week, showing a higher urinary flow rate (SMD=0.91; 95%CI [0.64, 1.18]; P<0.00001; I^2^=4%) with low heterogeneity (**Figure 11**). 3 studies [41, 56, 58] were treated over a week displayed a statistical significance (SMD=1.38; 95%CI [1.07, 1.70]; P<0.0001; I^2^=88%) with considerable heterogeneity (**Figure 12**). Sensitivity analysis we performed; heterogeneity disappeared when eliminating the study by Shengjia Zhao (2015). We speculated that the cause of high heterogeneity was the comparison of this study between PFMT and bromide with EA or electroacupuncture-free while no bromide was added in the other two studies. We also divided the studies into two subgroups based on the difference.

### 4.6. Urine Output

**Figure 13:**
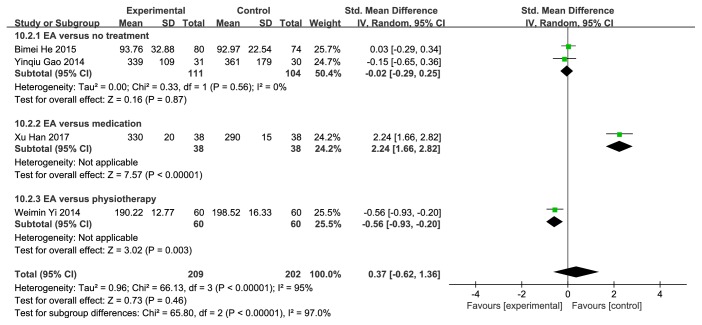
Forest plot of pooled estimation of the urine output (less than or equal to a week).

**Figure 14:**
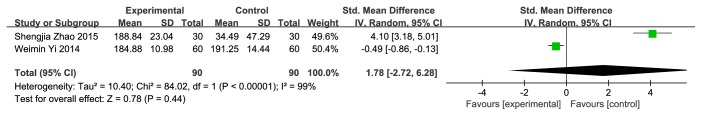
Forest plot of pooled estimation of the urine output (over a week).

There were 5 studies [41, 50–52, 56] which measured urine output according to healing period. 4 studies [41, 50–52] assigned to a group that treating less than or equal to a week showed a result of SMD=0.37 (95%CI [-0.62, 1.36]; P=0.46; I^2^=95%) (**Figure 13**). And 2 studies [41, 56] distributed to a group treating over a week showed a result of SMD=1.78 (95%CI [-2.72, 6.28]; P=0.44; I^2^=99%) (**Figure 14**). Both of two results were statistically nonsignificant.

### 4.7. Adverse Events

Only 4 studies [41, 42, 57, 60] reported adverse events. The most common adverse events were nausea and local hematoma. The study by Liang Chen (2007) stated no adverse events in the research. 1 study [41] reported that 9 of the 60 patients in experimental group had adverse effects. 6 got local hematoma and 3 got local muscle convulsion. 1 case of the 60 patients in control group got local hematoma; 1 study [57] stated a case of urinary system infection in the EA group and 2 cases in the control group which were irrelevant to the invention. Another study [42] reported no adverse events in the experimental group, 8 cases of nausea, and one dizziness in the control group, about 18%.

### 4.8. Publication Bias

**Figure 15:**
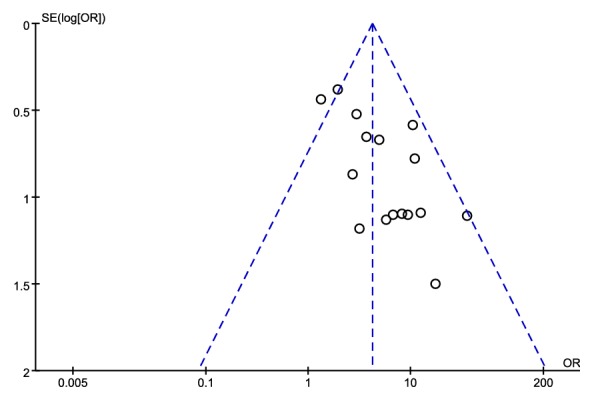
Funnel plot of the publication bias.

Publication Bias Evaluation on total therapeutic effect of EA (less than or equal to a week) was conducted using RevMan. The therapeutic effect was analyzed through funnel plots, which included 16 trials and 1382 objects. All of the included studies were from China and had small sample sizes. Results revealed that the distribution of included studies was asymmetric on both sides of the funnel plots, indicating that it may have publication bias in the therapeutic effect of EA (less than or equal to a week) (**Figure 15**).

### 4.9. Level of Evidence

Overall the quality of evidence accessed via GRADE for comparisons was very low to moderate; most were very low, limiting our confidence in trial findings. We rated few studies as having low risk of bias, and for utmost studies, we assigned evaluation of high risk of bias in at least one domain. High risk of bias was most frequently related to the domains of random, blinding, and selective reporting which caused reviewers to downgrade evidence by at least one level for each comparisons, except for one [41]. Attrition bias was rated as low risk that they provided complete data in the study or elaborated on the reasons for the loss. Other biases were all rated as low risk. Several comparisons showed substantial (I^2^>50%) heterogeneity, and the comparison of residual urine volume (over a week) had considerable heterogeneity of I^2^= 95%. Although most of the heterogeneity can be explained by healing period and interventions themselves, substantial heterogeneity was often significant enough to result in downgrading of the level of evidence. We were unable to examine the effect of study quality through a sensitivity analysis that we found only a study [41] was at low risk of bias. The quality of inconsistency remains poor in many studies. Most of them caused imprecision and publication bias due to their small sample size and did not report adverse events that downgraded evidence. Comparisons among studies were conducted directly that indirectness did not downgrade** (Table 2)**.

## 5. Discussion

The objective of this review is to summarize and evaluate the therapeutic effect and safety of EA treatment through the residual urine volume, time to first urination, bladder capacity, urinary flow rate, and urine output in patients with PUR. We included 23 studies, 1861 participants into the meta-analysis. Data showed significant heterogeneity of EA for comparison with controls such as medication, physical therapy, and no treatment.

Regarding our primary outcome of therapeutic effect of EA, 16 studies included, with statistically significant benefits of EA treatment for both less than or equal to a week and over a week as primary or an adjunct role. The result showed that EA as comparison with other treatments had a significantly higher total effective rate. Substantial heterogeneity existed; the level of evidence was very low to low, limiting our confidence of EA in PUR. The amount of residual urine volume suggested a visible reduction of EA rather than other treatments, accompanying with considerable heterogeneity and very low to low level of evidence. The advance of first urinating time was statistically significant with substantial heterogeneity while the evidence quality was very low to moderate. Improvement of bladder capacity and urinary flow rate illustrated statistically significant with very low to low level of evidence. However, the urine output measured by 5 studies [41, 50–52, 56] were statistically nonsignificant. Above comparisons were rated as very low to moderate level of evidence; most were very low and low. Outcomes described in the previous paragraph were lacking of credible evidence to show the effectiveness of EA in treating PUR. It is proved that the effect of EA on PUR is weak so far. Additionally, adverse reactions were reported descriptively. The most common adverse events were nausea and local hematoma, which were within the acceptable range.

Although the experimental group shows a significantly improved effectiveness in comparison to the control group, further research and studies are needed since most included studies are of low methodological quality. All studies were published in China with a risk of bias that prohibited clear conclusions. No multicentre or multiblinded studies existed. The sample size in many studies was small and reports of adverse reactions were affected. Only 4 studies reported adverse reactions, so additional large-scale clinical trials were needed before conclusions were reached. Adverse events reported from studies were limited, and within the reported adverse events, it can be concluded that the adverse events from EA were not as severe or serious as other controls [41, 42, 57, 60]. Even though acupuncture is free from the risk of serious adverse events [62], most serious adverse events can be prevented via mindful and hygienic administration and education.

The mechanism by which EA relieves PUR is not clear, but the optimistic effect is being confirmed by many studies. We have difficulty in drawing a definitive conclusion that EA is more effective than other treatments. EA has been widely used in China; it relieves pain for many patients safely and effectively [63, 64], and its mechanism and effect are worthy of our in-depth study.

Some limitations and deficiencies exist in the research. First, the follow-up data of this treatment to estimate the long-term efficacy is insufficient and requires more research to continue. Diversification of research interventions results in fewer studies comparing each intervention; accordingly, the sample size of each comparison also decreases. Second, some studies lack details of randomization sequence generation, allocation concealment, and blinding. Considerable heterogeneity among studies owing to different interventions and treating time have been handled with subgroup analyses and sensitivity analyses. Third, the analysis has not considered different types of needles or degree of operator experience, which may affect the findings and results. Without limits of nation or language, all included studies were conducted in China, potential publication bias might exist. Otherwise, very few negative results and unavailable data might cause bias as well. We did not compare the difference in efficacy of EA with usual care, medication, physical therapy, or no treatment or compare EA plus traditional treatment as an adjunct role with simple traditional treatment; due to the small number of studies included, it was hard to conclude an accurate result.

## 6. Conclusion

Overall, this systematic review and meta-analysis suggests that the effect based on the use of EA for improving the therapeutic rate, reducing residual urine volume, advancing first urinating time, and increasing bladder capacity and urinary flow rate with PUR is weak. EA has relatively fewer side effects in the treatment of PUR and is comparatively safe. However, the sample size of the very studies is not large enough and there is insufficient evidence of high quality. Therefore, large-scale, long-term RCTs with rigorous methodological quality are needed to clarify the role of EA in PUR. Further research is needed to understand long-term efficacy and the mechanism of action of the intervention.

## Figures and Tables

**Figure 2:**
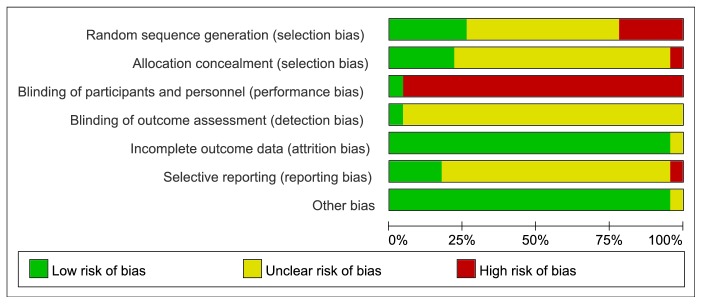
Risk of bias graph.

**Figure 3:**
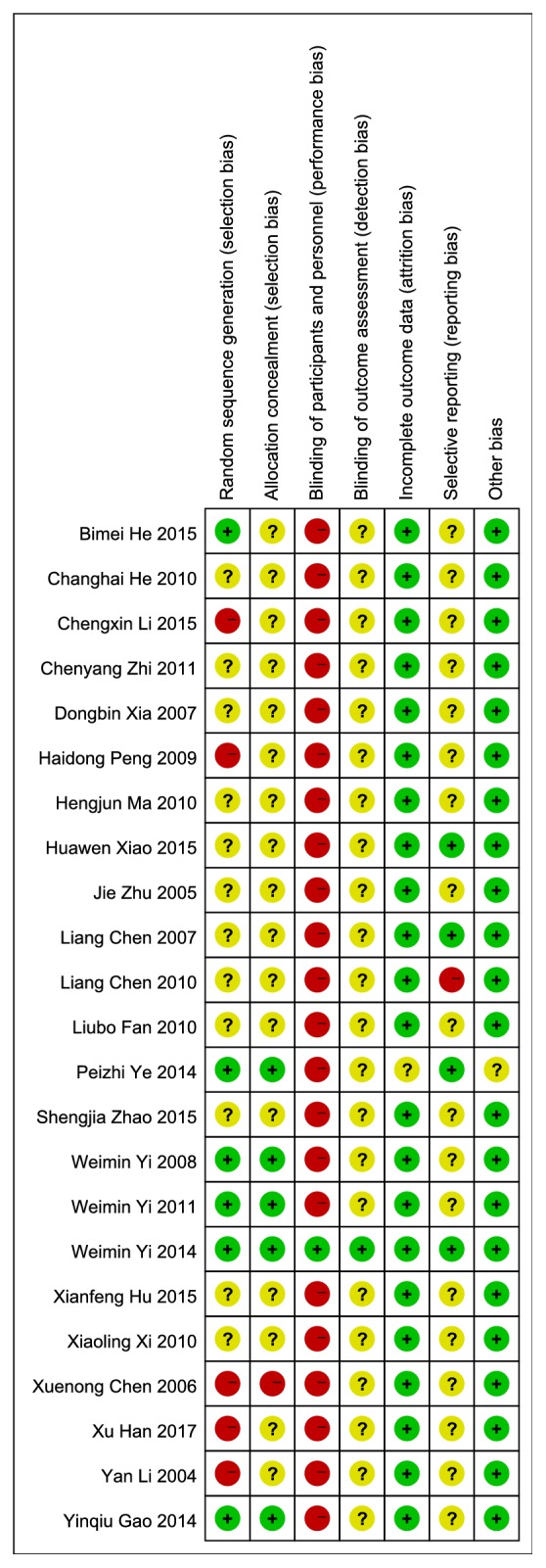
Risk of bias summary.

**Table 1:** Characteristics of the included studies.

Study	Number of participants	Finished number	Age y	Mean age y	Course of disease Y(m)	experiment	control	Duration	Outcomes (primary/secondary outcome)	BMI	Operation	Starting time
Weimin Yi2011	E:40 C:40	E:40 C:40	20-70	E:46.2±8.6 C:46.9±8.9		EA	VitaminB12	2w, 5 days a week	Therapeutic effect, residual urine volume	E:23.6±5.6 C: 24.2±6.1	Radical Hysterectomy	15^th^ day

Changhai He 2010	E:32 C:15	E:32 C:15		E:73.9 C:73.5		EA	Basic nursingr+Adrenergic *α*1 receptor blockade	4W,	Therapectic effect		Prostatectomy	6-8^th^ day

Jie Zhu 2005	E:53(m30,f23) C:57(m35,f22)	E:53 C:57	E:19-71 C:22-75	E:46 C:43		EA+moxibustion	neostigmine		Therapeutic effect		anal fistula operation, mixed hemorrhoid operation,anal fissure operation	

Weimin Yi2014	E:60 C:60	E:58 C:57	20-65	E:46.5±7.7 C:45.9±8.2		EA	SA	3W	Therapeutic effect, residual urine volume, bladder capacity, maximum urinary flow rate, urine output	E:24.3 ±3.4 C:25.1 ±3.3	radical hysterectomy	6^th^ day

Huawe Xiao 2015	E:50(m28,f22) C:50(m23,f27)	E:50 C:50	E:19-65 C:20-66	E: 42. 56± 11. 68 C:41.09± 12. 87	E: 8. 78±4. 56 C:9. 04±5. 37	EA	Tylox	3 days	Therapeutic effect		Anorectal surgery	

Liang Chen 2007	E:35 C:36	E:35 C:36				EA	No treatment		Therapeutic effect		anus operation	

Chenyang Zhi 2011	E:32(m17,f15) C:30(m11,f19)	E:32 C:30	24-65	E:47.5±17.5 C:43.5±19.5		EA	No treatment	30min	Therapeutic effect		anus operation	

Liang Chen 2010	E:30(m14,f16) C:30(m17,f13)	E:30 C:30				EA	No treatment		Therapeutic effect, first urination time			

Hengjun Ma 2010	E:60(m33,f27) C:60(m31,f29)	E:60 C:60	E:16-65 C:20-64	E:45.5 C:44.6		EA+Chinese medicine liquid	No treatment	3 days	Therapeutic effect		Mixed hemorrhoid surgery	

Weimin Yi2008	E:55 C:55	E:55 C:55	20-70	E:47.5±7.8 C:46.3±8.2		EA+basic nursing+TDP	Basic nursing+TDP	5 days, once a day	Therapeutic effect, residual urine volume, bladder capacity, maximum urinary flow rate	E:22.3 ±2.3 C:22.6 ±2.5	Extensive hysterectomy	8^th^ day

Xiaoling Xi 2010	E:31 C:31	E:31 C:31	26-65	44.4		EA+PFMT+basic nursing	PFMT+basic nursing	5 days, once a day	Therapeutic effect, residual urine volume		Extensive hysterectomy, pelvic lymph node dissection	6^th^ day

Chengxin Li 2015	E:33(m19,f14) C:34(m20,f15)	E:33 C:34	E:21-61 C:19-62	E:43±12 C:44±11		EA+PFMT+basic nursing	Basic nursing	2w, once a day	Therapeutic effect, residual urine volume, first urination time			

Xianfeng Hu 2015	E:42 C:42	E:42 C:42		E:48.5±10.4 C:47.9±12.4		EA+TCM nursing	Basic nursing	2w, once a day	Therapeutic effect, residual urine volume		Radical Hysterectomy	16^th^ day

Haidong Peng 2009	E:23 C:23	E:23 C:23	E:26-69 C:24-66	E:34 C:35	7-14d	EA+gingeer moxibustion	neostigmine	5 days, once a day	Therapeutic effect		Radical Hysterectomy	8-15^th^ day

Dongbin Xia 2007	E:40(m23,f17) C:40(m25,f15)	E:40 C:40	E:23-68 C:25-70	E:43.65 C:45.45		EA	neostigmine	20min	Therapeutic effect		anal fistula operation, mixed hemorrhoid operation, anal fissure operation	

Xuenong Chen 2006	E:30 C:30	E:30 C:30	E:35-69 C:33-68	E:39.51 C:39.5	2-5d	EA	neostigmine	5 days, twice a day	Therapeutic effect		Cervical cancer surgery	3-6^th^ day

Yan Li 2004	E:52 C:52	E:52 C:52	E:24-69 C:25-66	E:32.5 C:34.46	7-14d	EA	neostigmine	1w, once a day	Therapeutic effect		Extensive hysterectomy	8-15^th^ day

Shengjia Zhao 2015	E:30 C:30	E:30 C:30	E:60-74 C:60-75	E:63.7±3.2 C:64.2±2.7	7-14d	EA+PFMT+Bromides	PFMT+Bromides	20 days	Therapeutic effect, residual urine volume, bladder capacity, maximum urinary flow rate, urine output		Cervical cancer surgery	8-15^th^ day

Peizhi Ye 2014	E:74 C:33	E:74 C:33		E:46.92±13.69 C:46.26±12.89	E:94.12±65.36 C:85.34±73.67 d	EA+basic nursing	Basic nursing	10 days, once a day	Therapeutic effect, residual urine volume		Cervical cancer surgery	5^th^ day

Bimei He 2015	E:80(m45,f35) C:74(m39,f35)	E:80 C:74	E:20-76 C:19-76	E:45±16 C:48±16		EA	No treatment	30min	Therapeutic effect, first urination time, urine output		Spinal anesthesia	

Xu Han 2017	E:38(m28,f10) C:38(m26,f22)	E:38 C:38	E:46-78 C:44-78	E:59.8±9.14 C: 58.2±8.33		EA	Tamsulosin hydrochloride	3 day, once a day	Therapeutic effect, residual urine volume, first urination time, urine output		Hartmann, Dixon, Miles surgery	

Liubo Fan 2010	E:10(m6,f4) C:10(m5,f5)	E:10 C:10		E:57±14 C:55±11	E:9.3±1.5 C:9.1±3.7m	EA	Basic nursing	20 days, 5 times a week	Residual urine volume, maximum urinary flow rate		Pelvic fracture surgery	Half a year

Yinqiu Gao 2014	E:31(m10,f21) C:30(m9,f21)	E:31 C:28	18-65	E:55 ± 6 C:53 ± 11		EA	No treatment	30min	First urination time, urine output		Arthroscopic knee surgery	

**Table 2:** Level of evidence.

Variable	Effect(OR/MD)	95%CI	P	I2(%)	P(X2 test)	Statistical Method	Studies (N)	Sample size (N)	Level of evidence
Therapeutic effect (<=1w)	4.21	3.04, 5.83	<0.00001	33	0.09	Fixed effects models	16	1382	Very low
Therapeutic effect (>1w)	8.39	2.91, 24.25	<0.0001	61	0.02	Random effects models	7	565	Very low
EA versus medication	36.19	1.78, 734.32	0.02	83	0.003	Random effects models	3	187	Very low
EA versus physiotherapy	4.39	2.04, 9.42	0.0002	0	0.71	Random effects models	4	378	Low
Residual urine volume (<=1w)	-13.24	-13.70, -10.78	<0.00001	43	0.12	Fixed effects models	6	555	Low
Residual urine volume (>1w)	-2.02	-3.07, -0.98	0.0001	95	<0.00001	Random effects models	6	471	Very low
First urinating time	-0.92	-1.37, -0.46	<0.0001	79	0.0009	Random effects models	5	418	Very low
Minute	-1.24	-1.55, -0.93	<0.00001	35	0.22	Random effects models	3	197	Low
Hour	-0.36	-0.67, -0.04	0.03	—	—	Random effects models	1	154	Moderate
Day	-0.66	-1.15, -0.16	0.009	—	—	Random effects models	1	67	Very low
Bladder capacity (<=1w)	0.56	0.30, 0.83	<0.0001	0	0.63	Fixed effects models	2	230	Low
Bladder capacity (>1w)	0.92	0.61, 1.23	<0.00001	35	0.22	Fixed effects models	2	180	Low
Urinary flow rate (<=1w)	0.91	0.64, 1.18	<0.00001	4	0.31	Fixed effects models	2	230	Low
Urinary flow rate (>1w)	1.69	0.59, 2.79	0.003	88	0.0003	Random effects models	3	200	Very low
EA versus medication	2.65	1.95, 3.36	<0.00001	—	—	Random effects models	1	60	Very low
EA versus physiotherapy	1.06	0.70, 1.42	<0.00001	0	0.4	Random effects models	2	140	Very low

## Abbreviations

RCTs: Randomized controlled trials
EA: Electroacupuncture
PUR: Postoperative urinary retention
SMD: Standard mean difference
OR: Odds ratio
CI: Confidence interval
PRISMA: Preferred Reporting Items for Systematic Reviews and Meta-Analyses
CENTRAL: The Cochrane Central Register of Controlled Trials
CNKI: China National Knowledge Infrastructure Database
CBM: Chinese Biomedical Literature Database
WFDP: WanFang Digital Periodicals Database
KCI: Korean Citation Index
J-STAGE: Japan Science and Technology Information Aggregator, Electronic
RSCI: Russian Science Citation Index
ICS: The International Continence Society
MESH: Medical Subject Headings
GRADE: Grading of Recommendations, Assessment, Development and Evaluation
BMI: Body mass index.

## Supplementary Materials

Supplementary Materials Our study added PRISMA checklist (2009) which contained 27 items as a supplement to help us improve the reporting of systematic review and meta-analysis.
